# Enhanced diagnostic potential of CSPG4 in melanoma and nevi: a comparative study with PRAME, CDC7 and Ki67

**DOI:** 10.1002/path.6450

**Published:** 2025-07-23

**Authors:** Elias AT Koch, Christina Seidel, Ramona Erber, Michael Erdmann, Markus V Heppt, Stefan Schliep, Carola Berking, Jan Dörrie, Niels Schaft

**Affiliations:** ^1^ Department of Dermatology, Deutsches Zentrum Immuntherapie (DZI), CCC Erlangen‐EMN, Bavarian Cancer Research Center (BZKF), Uniklinikum Erlangen Friedrich‐Alexander‐Universität Erlangen‐Nürnberg (FAU) Erlangen Germany; ^2^ Institute of Pathology University of Regensburg Regensburg Germany

**Keywords:** CSPG4, PRAME, CDC7, Ki67, melanoma, benign nevus, dysplastic nevus, immunohistochemistry

## Abstract

Chondroitin sulfate proteoglycan 4 (CSPG4) is a promising target for melanoma immunotherapy, but its expression in benign melanocytic lesions and its diagnostic value remain unexplored. This study assessed CSPG4 expression in benign nevi (BN), dysplastic nevi (DN), and superficial spreading melanomas (SSM), comparing it with PRAME (PReferentially expressed Antigen in MElanoma) and evaluating the cell division cycle 7‐related protein kinase (CDC7) and the proliferation marker Ki67. Histological sections were stained using automated instruments, digitized, and analyzed using QuPath. Cohorts of BN, DN, and SSM were created, and positive cells/mm^2^ and H‐scores were determined. A total of 336 IHC stainings from 84 specimens were analyzed. CSPG4 expression was readily detected in SSM and was significantly stronger in DN (*p* = 0.005), with the highest intensity observed in BN (*p* < 0.001). PRAME showed the highest density of positive cells/mm^2^ in SSM, was significantly reduced in DN (*p* < 0.001), and was lowest in BN (*p* < 0.001). Ki67 expression was strong in SSM, moderate in DN (*p* = 0.62), and low in BN (*p* = 0.008). CDC7 expression was most intense in DN, less in SSM (*p* = 0.39), and weakest in BN (*p* = 0.002). ROC AUC values for SSM versus DN and SSM versus BN were 0.764 and 0.921 for CSPG4, 0.85 and 0.889 for PRAME, 0.735 and 0.742 for Ki67, and 0.425 and 0.767 for CDC7. While PRAME was the most reliable marker for differentiating DN and SSM, CSPG4 was superior for distinguishing BN from SSM due to its high expression in BN. However, CSPG4‐targeting therapies may trigger on‐target/off‐tumor effects due to its high expression in melanocytic nevi. © 2025 The Author(s). *The Journal of Pathology* published by John Wiley & Sons Ltd on behalf of The Pathological Society of Great Britain and Ireland.

## Introduction

Distinguishing benign nevi from malignant melanocytic neoplasms is a significant challenge for dermatopathologists. The primary method for histological diagnosis is based on conventional hematoxylin and eosin (H&E) staining and morphological examination of the characteristics of the lesion, such as pagetoid spread, cytological atypia, presence of dermal mitoses, asymmetry, lack of circumscription, impaired maturation, and hypercellularity. Commonly, immunohistochemistry (IHC) markers are used to confirm diagnosis.

One of these markers, PRAME (PReferentially expressed Antigen in MElanoma), raises a high degree of suspicion for melanoma when there is an increased cellular density of PRAME‐expressing cells [1, 2, 3]. Currently, PRAME is considered a reliable marker to support the diagnosis of melanoma, with diagnostic value shown for many different subtypes of melanoma, including uveal melanomas [4], metastatic melanomas [5], desmoplastic melanomas [6], as well as lentigo maligna [7, 8]. In a comparative evaluation of PRAME, Ki67, P16, and HMB‐45, Mert *et al* found that PRAME positivity was the most decisive factor in distinguishing between melanomas and nevi, in addition to morphological criteria [9].

However, subsequent studies have identified additional potential biomarkers, although the interpretation of these markers remains complex and lacks standardization. One such marker is CDC7 (cell division cycle 7), a serine–threonine kinase crucial for initiation of DNA replication and regulation of cell cycle progression. Its overexpression in melanoma cells offers potential as both a diagnostic marker and a therapeutic target. Clarke *et al* reported high CDC7 expression in nodular melanomas, atypical Spitz tumors, and superficial spreading melanomas (SSM) [10] and proposed that differences in CDC7 expression may facilitate in distinguishing melanomas from benign melanocytic lesions. However, no further validation of CDC7 as a diagnostic marker for melanoma has been reported.

Ki67 is a well‐established immunohistochemical marker, expressed during all active phases of the cell cycle (G1, S, G2, and mitosis), making it an excellent marker to assess cell proliferation. In melanoma, high levels of Ki67 expression can indicate a high proliferative index, which may correlate with more aggressive tumor behavior. In 2008, Ohsie *et al* identified Ki67 as the most useful marker for distinguishing benign from malignant melanocytic tumors [11]. According to their findings, less than 5% of cells are positive for Ki67 in benign nevi compared with an average of 13%–30% of cells in melanomas [11]. Vyas *et al* assessed the diagnostic utility of Ki67 staining for melanocytic lesions and reported that a low or negative Ki67 proliferation rate supports a benign diagnosis but does not exclude melanoma [12]. They recommended Ki67 as an ancillary test to support diagnosis, together with histopathology and other stains [12]. Furthermore, double stains of MART‐1/Ki67 have been reported to effectively differentiate melanoma from nevus [13]. However, Ki67 expression increases with tumor thickness in melanoma, suggesting reduced diagnostic utility in thin or borderline lesions [14].

Another target is chondroitin sulfate proteoglycan 4 (CSPG4), a transmembrane proteoglycan initially identified as an immunogenic tumor antigen on the surface of melanoma cells. It is also found in various partially differentiated precursor cells, such as oligodendrocyte progenitors in the central nervous system; developing mesenchymal cells in cartilage, muscle, and bone; as well as pericytes and smooth muscle cells in forming blood vessels. During the maturation process, CSPG4 is downregulated and may lose expression as cells undergo terminal differentiation [15, 16]. CSPG4 has also been shown to be expressed at the mRNA level in a primary melanocytic cell line (HEM1455), established from human neonatal foreskin, but at lower levels compared with a metastatic melanoma cell line (A2058) [17]. Protein expression, detected by IHC, was confirmed in melanoma, but no significant differences in CSPG4 expression between primary and metastatic melanoma were found [18]. CSPG4 further plays a role in the metastasizing process of melanoma [19] and is expressed on activated pericytes during angiogenesis in tumors and hypoxia [20, 21, 22], the latter facilitating targeting of tumor vasculature. CSPG4 acts as an oncogenic driver in melanoma, promoting growth and survival of malignant cells by signaling through various pathways [23], and therefore cannot be easily down‐regulated by the tumor to escape CSPG4‐targeted therapies. Therefore, CSPG4 is considered a prime tumor target antigen [24].

The H‐score (histochemical score) is a manual method for assessment of protein expression by IHC that combines staining intensity with the percentage of positive cells, resulting in a score ranging from 0 to 300 [25]. Through validation of both the extent and the intensity of staining, it provides a more nuanced assessment than the percentage of positive cells alone. Its application has been tested across various cancer types, underscoring its value in different pathological contexts [26, 27]. However, when employed manually, the H‐score is limited by significant inter‐observer variability, compromising its reproducibility [28]. Therefore, a standardized analysis tool is highly desirable, and several studies have focused on developing automated assessments of IHC slides to minimize bias [29, 30]. Standardized assessment of the H‐score can be performed using QuPath, an open‐source digital image analysis software increasingly used for various applications, including analysis of cell infiltration and IHC markers in solid cancer and lymphoma [31, 32, 33].

In this study, we utilized a standardized computer‐assisted assessment of four IHC markers (CDC7, CSPG4, Ki67, and PRAME) to differentiate between thin SSM, DN, and the common melanocytic nevus (also known as benign nevus, BN).

## Materials and methods

### Ethics statement

This study was approved by an independent research ethics committee of Friedrich‐Alexander‐Universität Erlangen‐Nürnberg, Germany (FAU; approval number 22‐368‐Br) and was conducted in accordance with the principles of the Declaration of Helsinki [34]. This retrospective analysis was based on 84 cases (28 BN, 28 DN, 28 SSM). All DN and BN were compound nevi. The tissue samples were obtained from the archives of the Unit for Dermatohistopathology, Department of Dermatology, Uniklinikum Erlangen, Germany. Patients were selected, and information was gathered from their medical and pathology records.

### Immunohistochemistry

**Figure 1:**
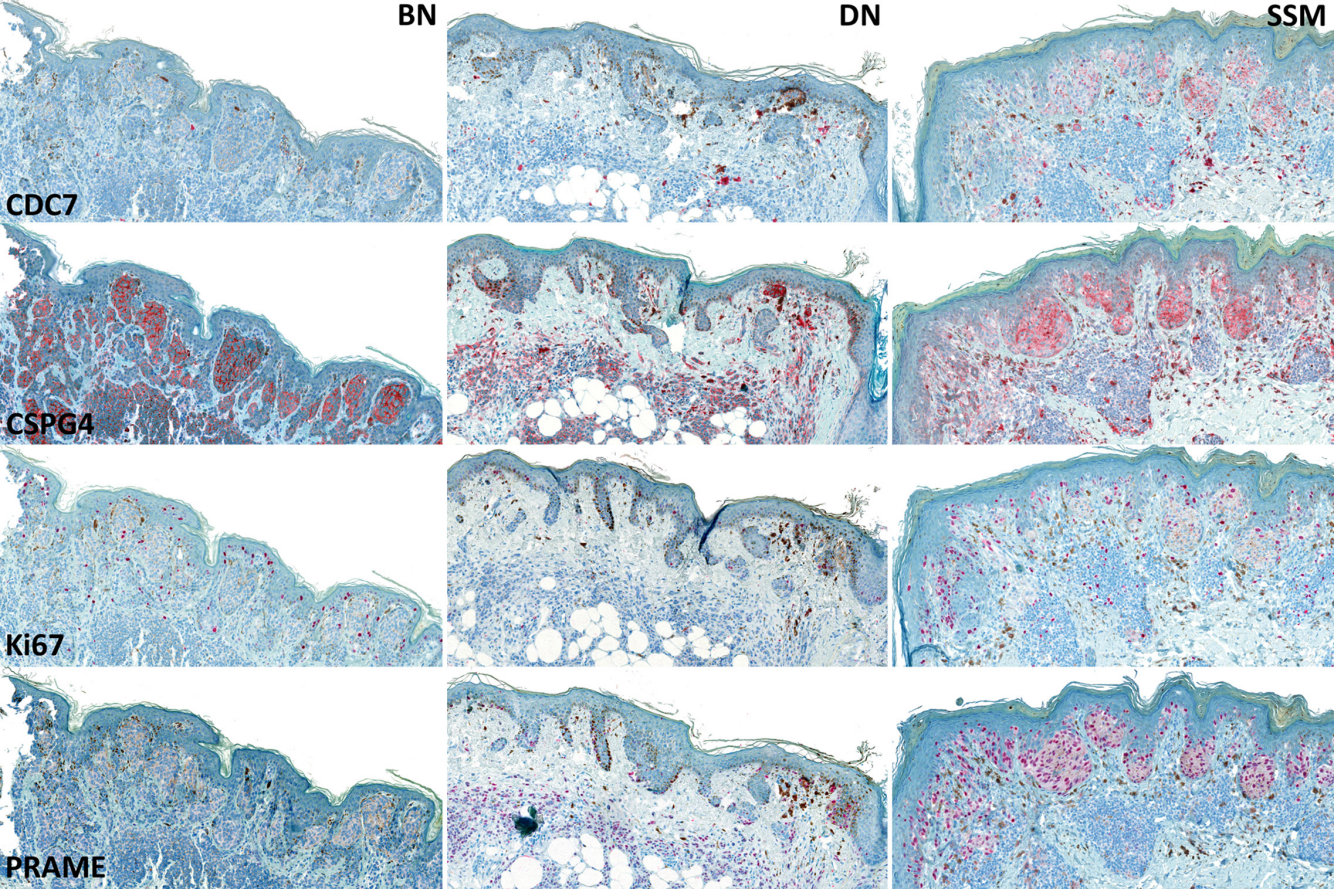
Column 1 shows a representative common benign nevus (BN), column 2 a dysplastic nevus (DN), and column 3 a superficial spreading melanoma (SSM). Row 1 depicts CDC7 staining, row 2 CSPG4 staining, row 3 Ki67 staining, and row 4 PRAME staining. Details of the staining protocols are provided in the Materials and methods section.

Commercially available antibodies were used for immunohistochemistry detection. For CDC7, a rabbit monoclonal antibody (clone number EPR20337; Abcam, Cambridge, UK) was used at a dilution of 1:100 with 32‐min incubation. For CSPG4, a rabbit monoclonal antibody (clone number EPR9195, Abcam) was utilized at a dilution of 1:500 with 36‐min incubation. For Ki67, a mouse monoclonal antibody (clone ZM67; Zeta Corporation, Sierra Madre, CA, USA) was used at a dilution of 1:200 with 32‐min incubation. For PRAME, a rabbit antibody (clone QR005; quartett GmbH, Berlin, Germany) was used at a dilution of 1:300 with 36‐min incubation. The antibodies for IHC slide staining were applied in a certified laboratory (Unit for Dermatohistopathology, Department of Dermatology, Uniklinikum Erlangen, Germany) using a fully automated BenchMark XT instrument (Roche Diagnostics, Rotkreuz, Switzerland). Fast Red chromogen (Roche Diagnostics, Rotkreuz, Switzerland) was used in the immunohistochemical staining process to avoid any interference from non‐specific cytoplasmic melanin pigment. An overview of the immunohistochemistry stainings is provided in Figure 1.

### Image data acquisition

**Figure 2:**
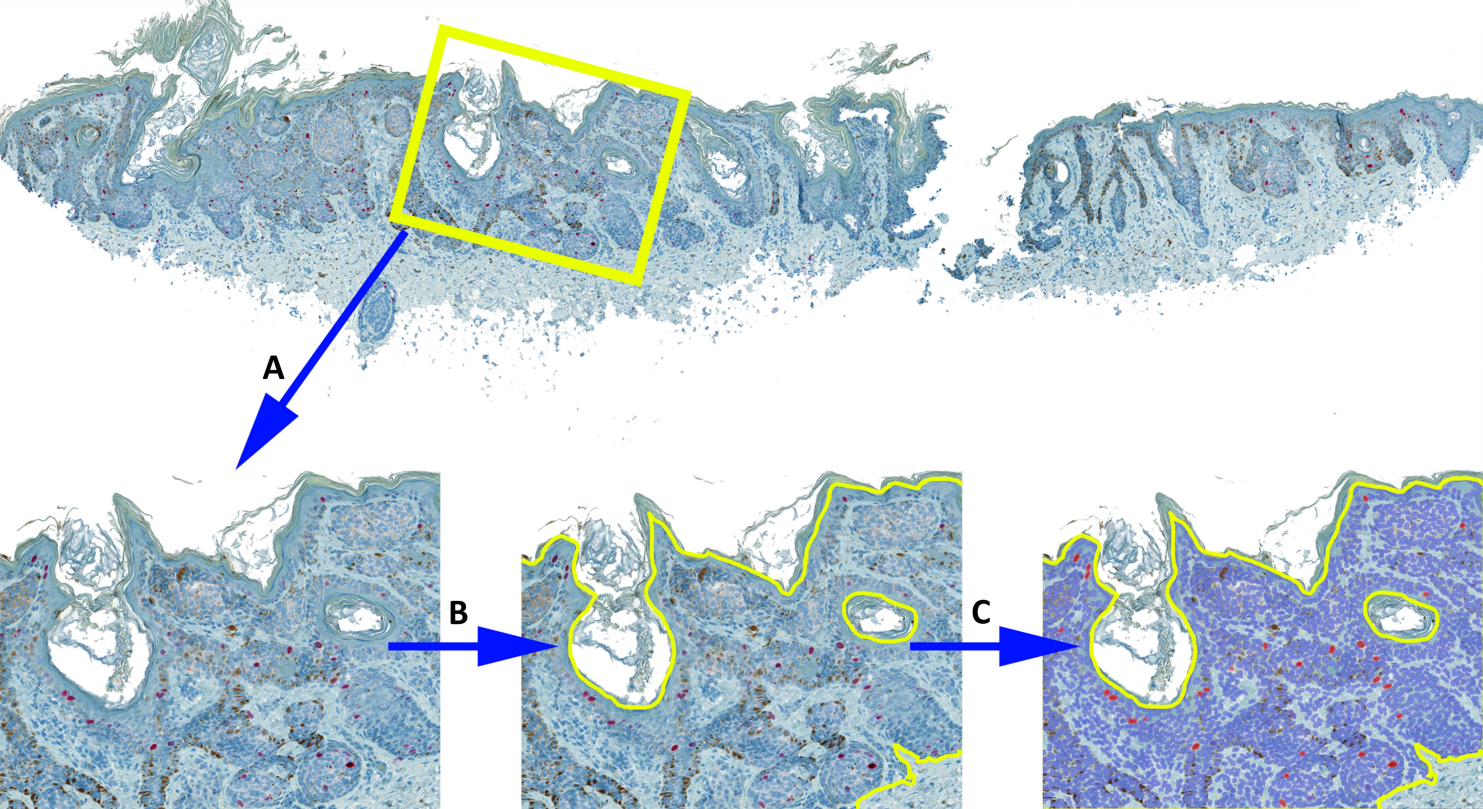
Simplified overview of the workflow steps in QuPath for Ki67 staining (melanoma). (A) Zoomed‐in area of a melanocytic lesion. (B) Schematic annotation for this image section (yellow frame). (C) Identification of Ki67‐positive cells (marked in red) and Ki67‐negative cells (marked in blue) within the annotated area (yellow frame), according to the QuPath script.

All slides were digitized using a Fritz digital microscope + scanner (PreciPoint, Garching, Germany) at 40× magnification, with a resolution of 0.23 μm/pixel. The IHC‐stained slides were analyzed using QuPath bioimage analysis software (version 0.5.1) [35], as previously described [3, 36]. In brief, for all stains, regions of interest (ROIs) that included the entire melanocytic lesion (both epidermal and dermal components) were manually annotated in QuPath. A script was written within the QuPath platform to detect positive cells for each stain (CDC7, CSPG4, Ki67, PRAME). All cells within the annotated area were identified to distinguish positive and negative cells (Figure 2). The number of positive cells/mm^2^ and the H‐score for all annotations were extracted from QuPath. The H‐score was automatically calculated by QuPath based on the positive and negative classification. QuPath determined the percentage of positive cells in three different staining categories (weakly positive, moderately positive, and strongly positive) and then multiplied these percentages by the corresponding category values (1, 2, or 3). The final score, obtained by summing these products, ranged from 0 to 300, with 0 signifying that all cells were negative and 300 indicating that all cells were strongly positive.

### Statistical analyses

Samples were grouped according to their diagnosis (SSM, DN, BN) and compared using Student's *t*‐test. A two‐sided *p* value of <0.05 was used to define statistical significance. Levene's test was used to assess the equality of variances across the different groups. If the *p* value was <0.05, indicating unequal variances, Welch's *t*‐test was performed. Additionally, the different groups (SSM versus DN, SSM versus BN, and SSM versus DN and BN) were classified using receiver operating characteristic (ROC) analysis and the area under the ROC curve (AUC). An ROC AUC score of >0.8 was considered good and >0.9 very good [37]. All statistical analyses were performed using IBM® SPSS Statistics (version 28.0.0.0; IBM, Armonk, NY, USA).

## Results

Overall, 336 IHC stainings of 84 different specimens were analyzed, including 28 (33.3%) SSM, 28 (33.3%) DN, and 28 (33.3%) BN. All DN and BN were compound nevi. The mean tumor thickness of SSM was 0.39 mm [standard deviation (SD) 0.15 mm]. Additional clinical features are summarized in Table 1.

**Table 1:** Baseline characteristics of the three groups of samples.

	BN (*n* = 28)			DN (*n* = 28)			SSM (*n* = 28)		
		Frequency	Percent		Frequency	Percent		Frequency	Percent
Location	Head	0	0	Head	3	10.7	Head	3	10.7
	Trunk	20	71.4	Trunk	19	67.9	Trunk	13	46.4
	Upper extremity	3	10.7	Upper extremity	3	10.7	Upper extremity	8	28.6
	Lower extremity	5	17.9	Lower extremity	3	10.7	Lower extremity	4	14.3
Sex	Female	17	60.7	Female	11	39.3	Female	15	53.6
	Male	11	39.3	Male	17	60.7	Male	13	46.4

	BN (*n* = 28)		DN (*n* = 28)		SSM (*n* = 28)	
	Mean	SD	Mean	SD	Mean	SD
Age (years)	48.9	14.1	49.3	16.8	57.7	14.1
Tumor thickness (mm)	N/A	N/A	N/A	N/A	0.39	0.15

BN, benign nevus; DN, dysplastic nevus; N/A, not applicable; SSM, superficial spreading melanoma; SD, standard deviation.

A significant difference in the number of positive cells/mm^2^ was observed between SSM and BN for all investigated proteins (CDC7, *p* = 0.011; CSPG4, *p* < 0.001; Ki67, *p* = 0.008; PRAME, *p* < 0.001) and between SSM and DN for CSPG4 (*p* = 0.005) and PRAME (*p* < 0.001), respectively. Further details are provided in Table 2 and supplementary material, Table S1 and Figure S1.

**Table 2:** Mean number of positive cells per mm^2^ and mean H‐score for BN, DN, and SSM.

		BN (*n* = 28)	DN (*n* = 28)	SSM (*n* = 28)
CDC7	Cells/mm^2^ mean (SD)	19.8 (35.9)	98.2 (122.9)	72.7 (97.9)
	H‐score mean (SD)	4.5 (2.3)	3.1 (2)	6.7 (5.8)
CSPG4	Cells/mm^2^ mean (SD)	2,080.4 (1,026.1)	958.6 (851.3)	392.7 (561.9)
	H‐score mean (SD)	26 (10.9)	12.2 (10.5)	5 (7.3)
Ki67	Cells/mm^2^ mean (SD)	157.3 (66.3)	143.4 (78.7)	294.6 (245.2)
	H‐score mean (SD)	3.9 (1.4)	3.1 (1.9)	6.6 (5.8)
PRAME	Cells/mm^2^ mean (SD)	25.4 (49.3)	48.8 (105.4)	426 (409.8)
	H‐score mean (SD)	0.4 (0.6)	0.7 (1.8)	7.5 (7.9)

BN, benign nevus; DN, dysplastic nevus; SSM, superficial spreading melanoma.

**Figure 3:**
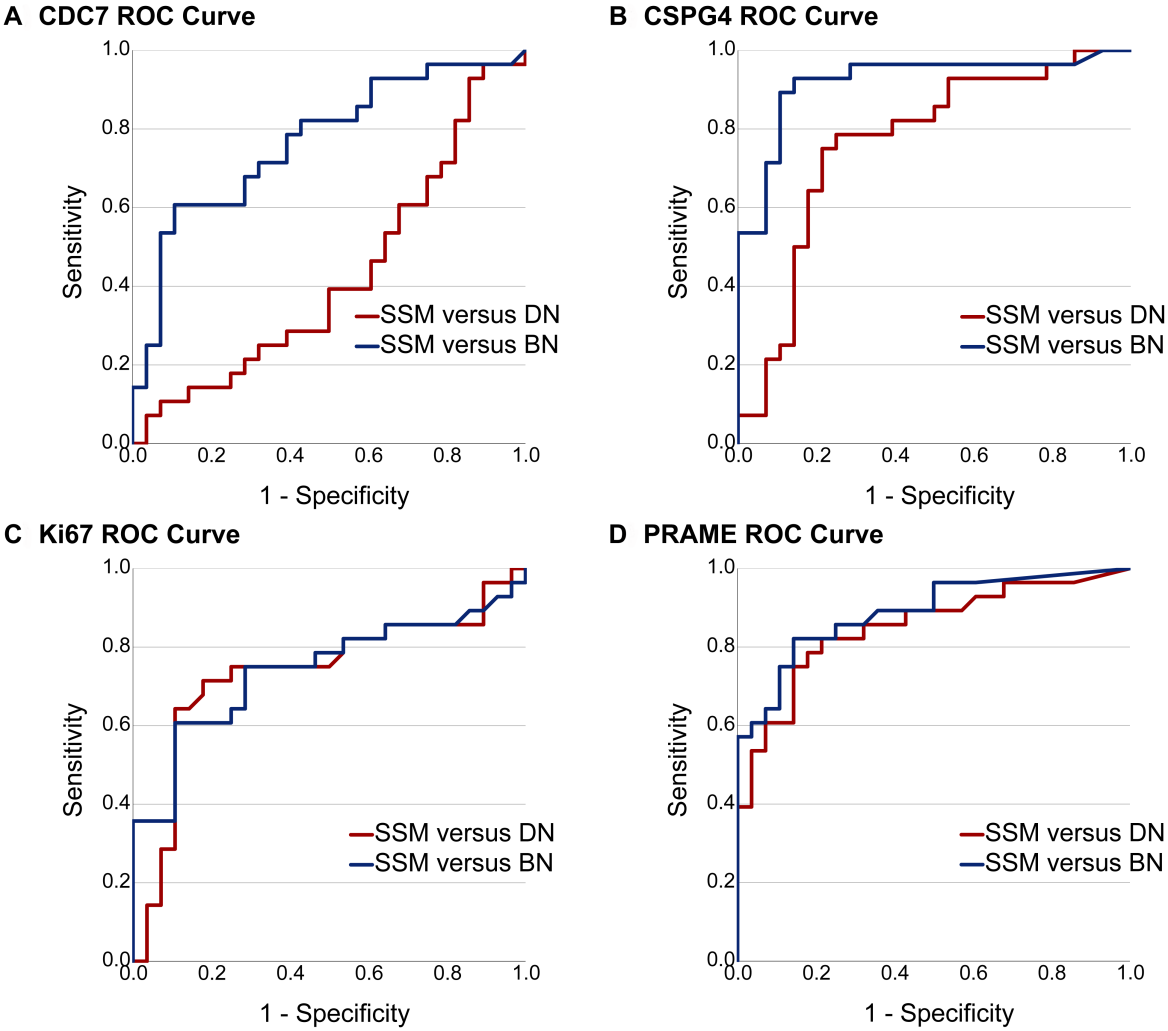
ROC curves for SSM versus DN (red) and SSM versus BN (blue). ROC AUC values for (A) CDC7: 0.425 (SSM versus DN) and 0.767 (SSM versus BN), respectively; (B) CSPG4: 0.764 and 0.921, respectively; (C) Ki67: 0.735 and 0.742, respectively; and (D) PRAME: 0.85 and 0.889, respectively.

The ROC AUC analysis of CDC7‐positive cells gave values of 0.767 for SSM versus BN and 0.425 for SSM versus DN. Notably, the classification between SSM and BN using CSPG4 showed a very high ROC AUC value of 0.921, while the ROC AUC value for SSM versus DN was 0.764. The ROC AUC values of Ki67‐positive cells were 0.735 for SSM versus DN and 0.742 for SSM versus BN. The ROC AUC values of PRAME‐positive cells were 0.889 for SSM versus BN and 0.85 for SSM versus DN. Further details are provided in Table 3 and Figure 3. The ROC AUC value for differentiation of DN and BN using the CDC7 stain was 0.797 (supplementary material, Figure S2). The ROC AUC values for SSM versus DN and BN are shown in supplementary material, Figure S3.

**Table 3:** ROC AUC values for the different biomarkers.

		ROC AUC	SE	*p* value	Asymptotic 95% CI	
					Lower bound	Upper bound
CDC7	SSM versus DN	0.425	0.078	0.333	0.273	0.577
	SSM versus BN	0.767	0.064	<0.001	0.642	0.893
CSPG4	SSM versus DN	0.764	0.066	<0.001	0.634	0.894
	SSM versus BN	0.921	0.040	<0.001	0.843	0.998
Ki67	SSM versus DN	0.735	0.072	0.001	0.594	0.875
	SSM versus BN	0.742	0.069	<0.001	0.607	0.878
PRAME	SSM versus DN	0.85	0.054	<0.001	0.739	0.950
	SSM versus BN	0.889	0.044	<0.001	0.800	0.974

BN, benign nevus; CI, confidence interval; DN, dysplastic nevus; ROC AUC, receiver operating characteristic area under the curve; SE, standard error; SSM, superficial spreading melanoma.

Regarding the healthy epidermis adjacent to nevi and melanomas, CSPG4 showed only slight cytoplasmic expression in common melanocytes of the skin (supplementary material, Figure S4). IHC analyses showed that CSPG4 was highly expressed in BN, with decreasing levels in DN and SSM. In contrast, PRAME showed the highest expression in SSM, with much lower levels in DN and BN. Notably, expression levels of PRAME and CSPG4 were similar in SSM. Ki67 and CDC7 exhibited distinct expression patterns across these tissue types.

## Discussion

In this study, we analyzed the expression of four different markers in BN, DN, and SSM, and assessed their potential to differentiate between benign and malignant melanocytic lesions of the skin. PRAME has become a widely used marker that facilitates melanoma diagnosis due to its association with many cellular processes relevant to tumor and metastasis development [2, 3, 9, 38]. The ROC AUC value for PRAME‐positive cells/mm^2^ in distinguishing DN and SSM is consistent with our previous study (ROC AUC = 0.866), confirming the reproducibility of the QuPath‐based method [3]. PRAME remains the most reliable marker for distinguishing DN from malignant melanocytic tumors, outperforming the other evaluated markers (CDC7, CSPG4, and Ki67). However, CSPG4 showed extensive expression in BN and significantly lower levels in SSM, contrary to expectations. Furthermore, CSPG4 was superior to PRAME in differentiating between BN and SSM, making CSPG4 a promising candidate for further investigation. Nevertheless, the average CSPG4 expression in SSM was still high and comparable to PRAME expression.

To date, CSPG4 expression had only been investigated in primary and metastasized melanoma tissues. In one study, in 53 metastases excised from 34 melanoma patients, the most consistently and strongly expressed antigen was CSPG4, which was present in 95% of the melanoma specimens and was expressed by a high proportion of cells within each specimen [39]. Another study demonstrated that in 26 out of 30 melanomas, more than 90% of the cells were positive for CSPG4 [40]. In addition, similar levels of CSPG4 expression were observed in melanoma metastases [39, 41].

To our knowledge, CDC7 has not been previously validated or used in clinical practice for the diagnosis of melanoma. In our study, analysis of CDC7 expression showed the highest number of positive cells/mm^2^ in DN, whereas the H‐score was lower in DN compared with SSM and BN. This demonstrates that more cells in DN express CDC7, but with a lower intensity. Regarding the expression of Ki67, the lowest number of positive cells/mm^2^ and H‐score were seen in DN, comparable to the levels in BN. This suggests that the cells in DN are not actively cycling but are more likely in the early S‐phase, during which CDC7 controls the initiation of DNA replication [42, 43]. High CDC7 expression indicates a potential for DNA replication and cell division, even if the cells are not actively proliferating, as reflected by low Ki67 expression. In this context, CDC7 may serve as a marker for differentiating between DN and BN. However, further studies are necessary to confirm its diagnostic utility.

Moreover, it is known that Ki67 expression is higher in melanoma than in benign melanocytic lesions. However, the interpretation of Ki67 is not standardized and has several limitations, as Ki67 may stain other proliferative cells [9, 12, 44]. Single‐stain analyses of Ki67 for melanoma diagnosis have reported an optimal cut‐off of 5%, with a positive predictive value of 62.5% sensitivity and 76.1% specificity [9]. In another study from two institutions, one reported a sensitivity of 90.9% and a specificity of 40% for the diagnosis of melanoma, while the other institution reported a sensitivity of 71.4% and a specificity of 56% [12]. Double staining with Ki67 and Melan A or MART1 can improve the diagnostic accuracy, likely due to the ability to distinguish Ki67‐positive melanocytes from other proliferating Ki67‐positive cells [13, 45]. For example, Ki67/MART1 double staining misclassified 2.3% of melanomas and 7.5% of nevi [13]. Another double‐stain study showed similar results, in which Ki67/MART1 double staining misclassified 4.1% of melanomas and 7.7% of nevi and reported a low cut‐off value of 1.6% for the Ki67/MART1 index [46]. Our study is in line with previous single‐stain analyses of Ki67, which supports the conclusion that Ki67 is helpful as an ancillary marker to support diagnosis, but it should be used with caution as a single stain [9, 12].

In addition to the potential diagnostic utility of CSPG4, a number of CSPG4‐targeting strategies, such as CSPG4‐specific monoclonal antibodies [47, 48, 49], radio‐immunoconjugates [50, 51, 52], chemotactic conjugates [53], or immunotoxins [54, 55, 56, 57, 58, 59, 60, 61], have already been previously applied in animal models and melanoma patients, with partially promising results [62]. Additionally, CSPG4‐specific chimeric antigen receptors (CARs) exerted potent cytotoxicity in response to various CSPG4‐expressing tumors, such as melanoma, breast cancer, mesothelioma, glioblastoma, and osteosarcoma [63, 64, 65, 66, 67, 68, 69, 70, 71], in animal models and *in vitro*. *In vivo*, the administration of CSPG4‐specific CAR‐T cells to mice bearing melanoma cells significantly stunted tumor growth and improved overall survival [65]. The same study described the non‐reactivity of CSPG4‐specific CAR‐T cells to normal tissues expressing detectable CSPG4 RNA, but without detectable CSPG4 protein expression [65]. Furthermore, CSPG4‐specific CAR‐T cells did not exert any significant cytotoxicity towards primary epithelial cell lines from prostate, lung, and kidney [65].

Considering the findings of our study, on‐target/off‐tumor reactions emanating from cellular therapies, such as CAR‐T cells specific for CSPG4, on melanocytic nevi can be expected. However, since melanocytic skin lesions are not essential tissues, these may be considered manageable from a medical point of view. Our observation that CSPG4 is not expressed on the membrane of melanocytes adjacent to a melanocytic lesion makes it unlikely that side effects will occur against healthy melanocytes in the skin or other tissues, unlike what has been observed with gp100‐specific immunotherapies [72]. Nevus cells in melanocytic lesions exhibit a different differentiation stage or altered maturation compared with normal melanocytes of the skin and may harbor genetic changes that can result in altered CSPG4 expression, as observed with anti‐BRAF immunostaining [73, 74]. Nevertheless, when developing a CAR specific for CSPG4, it should be considered that CSPG4 expression is even higher in nevi than in melanoma cells. This could present an opportunity to monitor the efficacy of the CSPG4‐targeted therapy in easily accessible nevi. Additionally, this approach may facilitate re‐isolation of CAR‐T cells to assess their functional state after antigen encounter.

This study faced some limitations: The blinding process was compromised, since the investigator could identify the diagnoses based on the overall morphology of the samples. Additionally, the identification of melanocytic cells was based solely on morphological characteristics, without the aid of counterstains for melanocytic markers like Melan A or SOX‐10. This introduces the risk of misidentifying some cells, especially in the dermis, where melanocytic cells may be mistakenly classified as immune cells or endothelial cells. This includes in particular that Ki67 was applied as a single stain without double staining, which stains not only melanocytes but also potentially proliferative keratinocytes in the epidermis and immune cells in the dermis.

In conclusion, PRAME immunostaining was the most reliable marker for differentiating between DN and SSM. CSPG4 exhibited an unexpectedly high level of expression in BN and was superior to PRAME in differentiating between BN and SSM. Furthermore, CSPG4‐targeted therapies may potentially lead to on‐target/off‐target reactions. Additionally, CDC7 may serve as a marker for distinguishing DN from BN. Importantly and counterintuitively, strong CSPG4 expression in nevi does not indicate malignancy, particularly when other tumor markers are absent.

## Author contributions statement

EATK conceived the original idea. EATK, JD and NS conceptualized the project. CS and EATK performed the image data acquisition. EATK performed the statistical analysis. EATK, JD and NS wrote the first draft of the manuscript. All authors revised the manuscript and provided critical feedback to shape the research and manuscript. All authors agreed to the final version of the manuscript.

## Supporting information


**Figure S1.** Expression of CSPG4 in comparison to other melanoma markers
**Figure S2**. ROC curve of CDC7 comparing DN (dysplastic nevus) with BN (benign nevus)
**Figure S3**. ROC curves of (A) CDC7, (B) CSPG4, (C) Ki67, and (D) PRAME comparing SSM (superficial spreading melanoma) with DN (dysplastic nevus) and BN (benign nevus)
**Figure S4**. H&E‐stained histological sections of healthy skin adjacent to a melanocytic lesion
**Table S1**. Statistical comparisons of biomarker expression between the different groups

## Data Availability

All data presented in this paper are available upon request from the corresponding author.
