# Supplementary material for: Enhanced diagnostic potential of CSPG4 in melanoma and nevi: a comparative study with PRAME, CDC7 and Ki67
Source: J Pathol. 2025 Jul 23;267(1):69–78. doi: 10.1002/path.6450 (PMC12337816; doi:10.1002/path.6450)
Supplement: Supplementary file 1 — Figure S1. Expression of CSPG4 in comparison to other melanoma markers Figure S2. ROC curve of CDC7 comparing DN (dysplastic nevus) with BN (benign nevus) Figure S3. ROC curves of (A) CDC7, (B) CSPG4, (C) Ki67, and (D) PRAME comparing SSM (superficial spreading melanoma) with DN (dysplastic nevus) and BN (benign nevus) Figure S4. H&E‐stained histological sections of healthy skin adjacent to a melanocytic lesion Table S1. Statistical comparisons of biomarker expression between the different groups [file PATH-267-69-s001.docx]

**Enhanced diagnostic potential of CSPG4 in melanoma and nevi: a comparative study with PRAME, CDC7 and Ki67**

EAT Koch *et al.* *J Pathol* <https://doi.org/10.1002/path.6450>

**Supplementary Figures S1–S4**

**Supplementary Table S1**


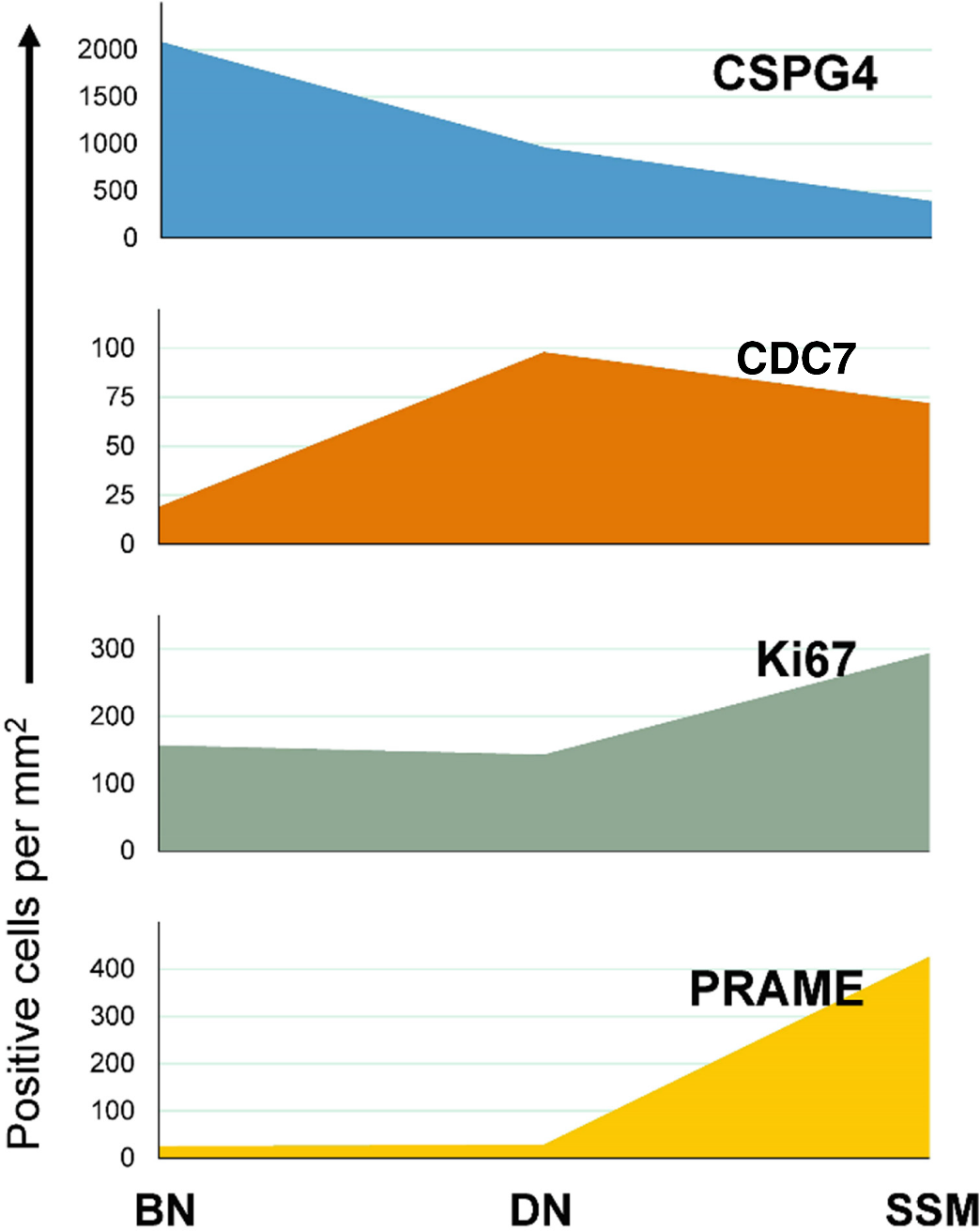


**Figure S1.** Expression of CSPG4 in comparison to other melanoma markers. Unexpectedly, CSPG4 expression in benign nevi (BN) exceeded that in dysplastic nevi (DN) and in superficial spreading melanoma (SSM), in contrast to CDC7, Ki67, and PRAME.


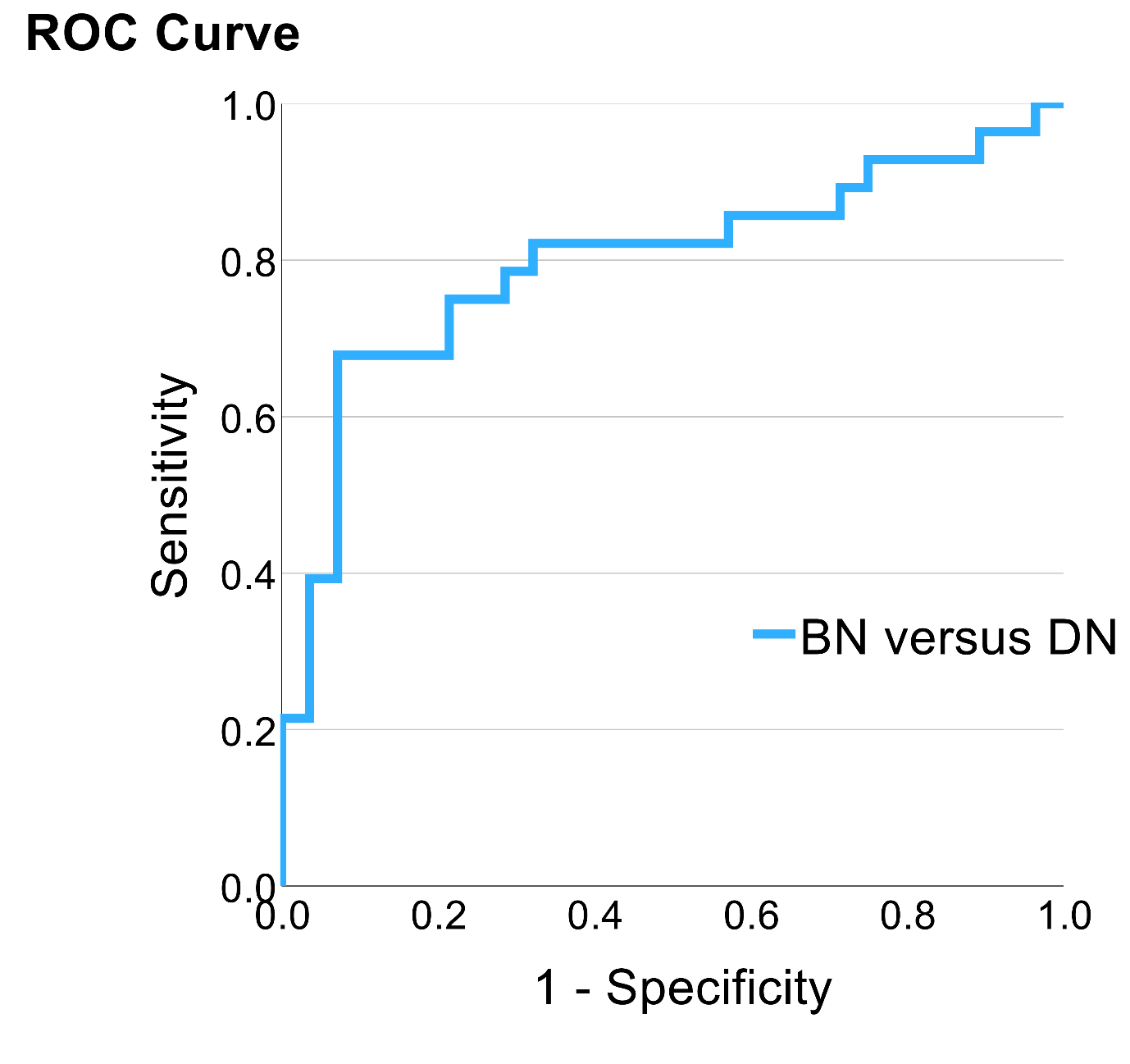


**Figure S2.** ROC curve for CDC7 comparing DN (dysplastic nevus) with BN (benign nevus). ROC AUC = 0.797 (SD 0.063; *p* < 0.001).


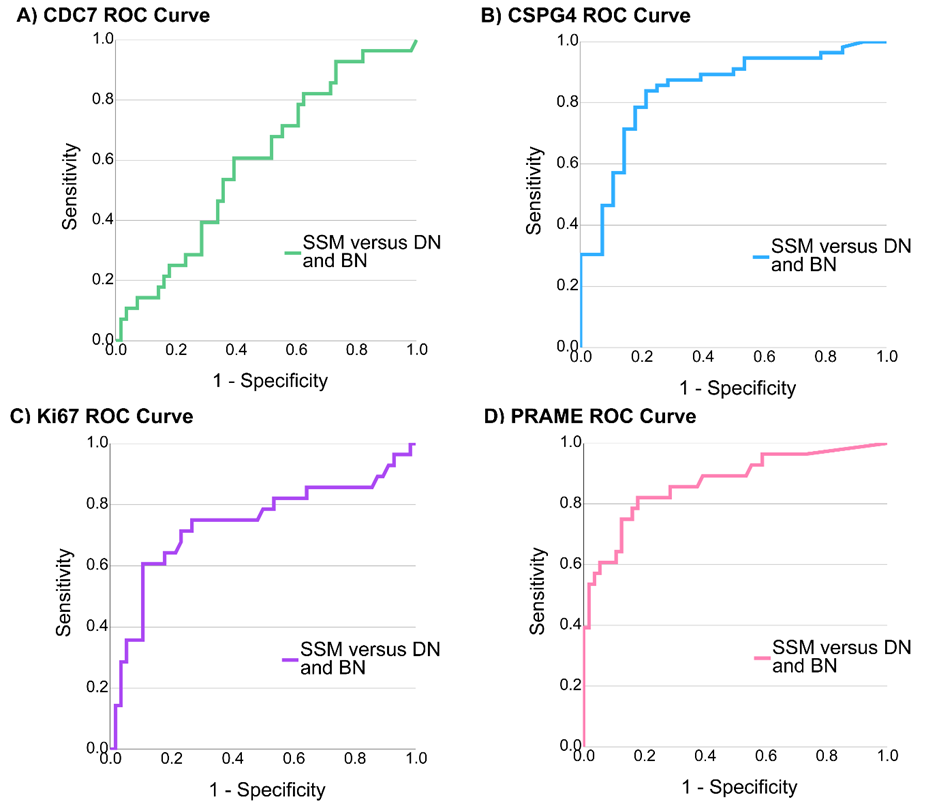


**Figure S3.** ROC curves of (A) CDC7, (B) CSPG4, (C) Ki67, and (D) PRAME comparing SSM (superficial spreading melanoma) with DN (dysplastic nevus) and BN (benign nevus). ROC AUC values: (A) 0.596 (SD 0.064; *p* < 0.133); (B) 0.842 (SD 0.046; *p* < 0.001); (C) 0.739 (SD 0.065; *p* < 0.001); and (D) 0.87 (SD 0.044; *p* < 0.001), respectively.


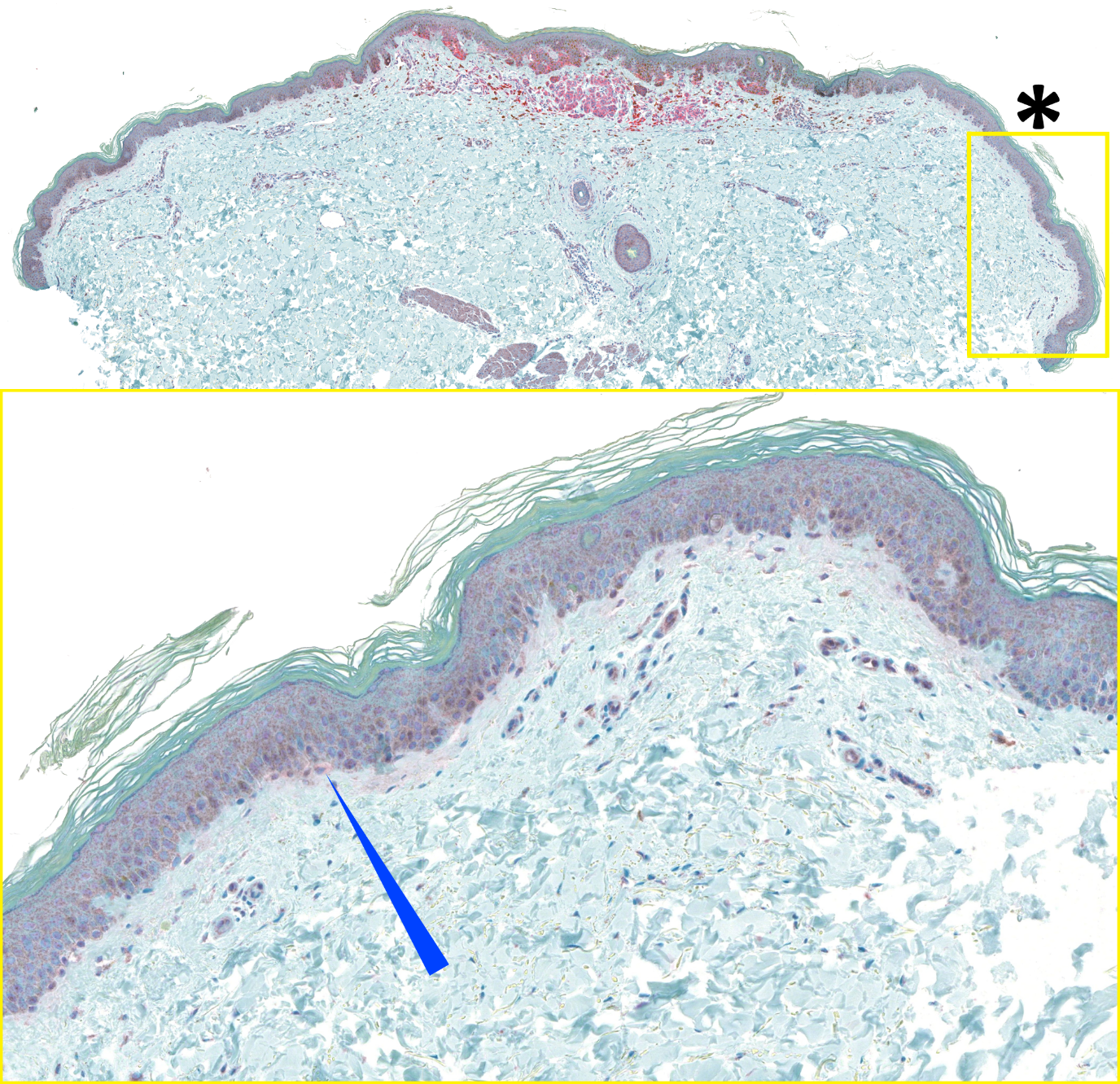


**Figure S4.** H&E-stained histological sections of healthy skin adjacent to a melanocytic lesion. CSPG4 exhibits slight cytoplasmic expression in common melanocytes of the basal layer of the epidermis (blue arrow). The top side of the yellow rectangle, indicated by the asterisk, has a length of 560 µm.

**Table S1.** Statistical comparisons of biomarker expression between the different groups.

| **Biomarker** | **Group comparison** | **Levene's Test** | **Student’s *t-*test / Welch’s *t*-test**  **(two sided, *p*)** | **Equal variances assumed** |
| --- | --- | --- | --- | --- |
| **CDC7** | SSM versus DN | 0.47 | 0.39 | Yes |
|  | SSM versus BN | < 0.001 | 0.011 | No |
|  | DN versus BN | < 0.001 | 0.002 | No |
| **CSPG4** | SSM versus DN | 0.29 | 0.005 | Yes |
|  | SSM versus BN | 0.005 | < 0.001 | No |
|  | DN versus BN | 0.133 | < 0.001 | Yes |
| **Ki67** | SSM versus DN | 0.14 | 0.62 | Yes |
|  | SSM versus BN | 0.015 | 0.008 | No |
|  | DN versus BN | 0.067 | 0.342 | Yes |
| **PRAME** | SSM versus DN | < 0.001 | < 0.001 | No |
|  | SSM versus BN | < 0.001 | < 0.001 | No |
|  | DN versus BN | 0.84 | 0.289 | Yes |

SSM (superficial spreading melanoma), DN (dysplastic nevus), and BN (nevus cell nevus).
